# Presenting evidence-based health information for people with multiple sclerosis: the IN-DEEP project protocol

**DOI:** 10.1186/1472-6947-12-20

**Published:** 2012-03-16

**Authors:** Sophie Hill, Graziella Filippini, Anneliese Synnot, Michael Summers, Deirdre Beecher, Cinzia Colombo, Paola Mosconi, Mario A Battaglia, Sue Shapland, Richard H Osborne, Melanie Hawkins

**Affiliations:** 1 Centre for Health Communication and Participation, Australian Institute for Primary Care and Ageing, La Trobe University, Melbourne, Australia; 2 Cochrane Multiple Sclerosis and Rare Diseases of the Central Nervous System Review Group, Unit of Neuroepidemiology, Fondazione IRCCS Istituto Neurologico, C. Besta, Via Celoria, Milan, Italy; 3 MS Australia, The Nerve Centre, Melbourne, Australia; 4 Laboratory for medical research and consumer involvement, Istituto di Ricerche Farmacologiche Mario Negri, Via La Masa, Milan, Italy; 5 Associazione Italiana Sclerosi Multipla, Via Operai, Genoa, Italy; 6 MS Australia, Perth, Australia; 7 Public Health Innovation, Population Health Strategic Research Centre, School of Health and Social Development, Deakin University, Melbourne, Australia

**Keywords:** Evidence-based patient information, Consumer involvement, Knowledge translation, Multiple sclerosis, Internet, Self-management, Preferences

## Abstract

**Background:**

Increasingly, evidence-based health information, in particular evidence from systematic reviews, is being made available to lay audiences, in addition to health professionals. Research efforts have focused on different formats for the lay presentation of health information. However, there is a paucity of data on how patients integrate evidence-based health information with other factors such as their preferences for information and experiences with information-seeking. The aim of this project is to explore how people with multiple sclerosis (MS) integrate health information with their needs, experiences, preferences and values and how these factors can be incorporated into an online resource of evidence-based health information provision for people with MS and their families.

**Methods:**

This project is an Australian-Italian collaboration between researchers, MS societies and people with MS. Using a four-stage mixed methods design, a model will be developed for presenting evidence-based health information on the Internet for people with MS and their families. This evidence-based health information will draw upon systematic reviews of MS interventions from The Cochrane Library. Each stage of the project will build on the last. After conducting focus groups with people with MS and their family members (Stage 1), we will develop a model for summarising and presenting Cochrane MS reviews that is integrated with supporting information to aid understanding and decision making. This will be reviewed and finalised with people with MS, family members, health professionals and MS Society staff (Stage 2), before being uploaded to the Internet and evaluated (Stages 3 and 4).

**Discussion:**

This project aims to produce accessible and meaningful evidence-based health information about MS for use in the varied decision making and management situations people encounter in everyday life. It is expected that the findings will be relevant to broader efforts to provide evidence-based health information for patients and the general public. The international collaboration also permits exploration of cultural differences that could inform international practice.

## Background

### Evidence-based health information for patients

Systematic reviews represent the highest level of evidence of the effectiveness of health care interventions [1]. Historically, it has been clinicians who have used evidence from systematic reviews of controlled trials to stay abreast of current research and inform their practice [2]. Increasingly, however, evidence-based health information, including systematic reviews, is also being provided to lay audiences [3].

A recognised source of high quality evidence is the Cochrane Collaboration [4]. Cochrane systematic reviews summarise evidence from trials on the effects of treatments (medicines, surgery, rehabilitation), and behavioural and informational interventions. These reviews are the most rigorous summary of the evidence available. They are kept up-to-date, with new evidence added every few years [4]. In Australia, as in many parts of the world, Cochrane systematic reviews are available free to the public because of a nationally-funded subscription to The Cochrane Library [5]. In Italy, Italian translations of selected plain language summaries of Cochrane reviews are available free to the public on the PartecipaSalute web site [6] and in the "SM Italia" journal of the Associazione Italiana Sclerosi Multipla (AISM).

Research has shown that evidence-based health information may improve people's knowledge, capacity to manage their health, and their health literacy [7,8]. An overview of more than 50 systematic reviews demonstrated a range of positive effects that may stem from informing and educating people about their health, and involving them in the management of their health [7]. The authors concluded that informational interventions, in conjunction with professional advice, can improve people's health literacy: effects include improvement in health knowledge and recall, reduced anxiety, improved symptom management, and an increased sense of empowerment. Examples of interventions that were shown to improve patient health literacy include well-designed and written health information and educational support materials, computer-based and Internet-based information resources, and targeted mass-media approaches for specific population groups [7].

Research into accessible and usable formats for communicating evidence to lay audiences has flowed from these initiatives and has focused primarily on how to present evidence-based information clearly and unambiguously [9,10]. This is vital in order to avoid the non-transparent framing of risk and benefit information, and the associated consequences of unintended or intended manipulation of risk information [11,12]. The results of such studies are promising. For example, a randomised controlled trial of a drug leaflet advertisement that included a fact box about the medicine (quantifying outcomes with and without the drug) showed that the intervention improved patients' knowledge of drug benefits and side effects, resulted in better choices of drugs for symptoms, and improved patients' ability to make a realistic assessment of benefits, compared with a drug advertisement without the fact box [13].

### Health information and people with multiple sclerosis

Developments in the delivery of evidence-based health information have critical implications for providing information to people with multiple sclerosis (MS). Research has demonstrated that information plays an important role for people with MS to help them understand their diagnosis and treatments, and for self-management education [14,15]. MS is a complex inflammatory disease of the central nervous system, characterised by progressive neurological dysfunction, for which both a cure and the cause remain elusive. MS affects about 2.5 million individuals throughout the world [16]. Diagnosis typically occurs between the ages of 20 and 40. People with MS are high users of the full range of health services and, in particular, of medicines [17]. Despite the fears and anxieties that may arise from learning about the disease, the treatments, and self-management strategies, Wollin et al. [14] conclude that information is a critical component for informed decision making, problem solving, and self-determination.

The advent of disease-modifying drugs for MS means that people are actively seeking information about new treatments, and earlier attitudes of hopelessness are changing [18]. German research has found that people with MS were seeking more information than was provided at the time of their diagnosis, including information about potential treatments [18]. Later studies identified that people were requesting active roles with regard to medical decision making, and that they understood complex health and research information [19,20]. This finding opens up the possibility of shared decision making between patients and doctors about treatment options and health management.

Concurrently, the information-seeking landscape has changed dramatically with the addition of the Internet, which can facilitate greater opportunities for shared decision making. While medical professionals and MS societies have long been the preferred and trusted sources of health information, the Internet is gaining increasing prominence, particularly amongst younger people [21]. Studies conducted in Israel and the US found that between 64% and 82% of people with MS seek health information online [21,22].

In many countries, non-government organisations such as MS societies play a critical role in providing information for people with MS [23] and are using their web sites to provide information to people or to refer them to other high-quality sources. The move to online technologies reflects consumer demand and a population need to foster confident and skilled users of information. It is also fulfilling non-government organisations' roles to promote a higher level of client and community engagement in health care and service models, and to support policy for public participation in the health service system [24,25]. Users of non-government web sites are not only consumers. Health professionals play two important roles in people's Internet-based health-information seeking: 1) they may work in partnership with patients and patient groups to obtain and analyse information, and 2) they can guide Internet users toward reliable health information web sites [26]. The development of high quality, evidence-based health information on non-government sites must therefore accommodate these different user groups and their needs.

### Making evidence meaningful for people

Our assertion is two-fold. First, that people with MS who read summaries of systematic reviews for health information may need additional documented support or advice to help them understand how the information can be applied to their personal context [14,27]. This documentation may also support people to use the information in the systematic reviews when making decisions and judgements with their doctors about treatment preferences or self-management options that will work for them. There is a paucity of data about the experiences people have when needing to assess evidence-based information and integrate it into their own context and value system. The second part of our assertion addresses the process of developing complex interventions, such as health information services for patients. In order to supply a service that is applicable to a population group, the information and other needs of those individuals must first be understood. The next steps are to identify the evidence base, build or refine the theoretical concepts, then evaluate the processes and outcomes for implementation, before trialling the intervention [28].

We know that many patients and family members want to understand the evidence that supports treatment options [10]. They also want to know how the research relates to them individually [29], as well as the implications of the findings for their future treatment choices and management options. This creates a challenge for services that provide health information based on research because that information may not be sufficient for an individual to make the connection between the research and their own circumstances. Further information may need to be provided by the health information service in order for people to be able to make judgements about the appropriateness of a treatment for their own context. Furthermore, as new research is conducted, people may need to understand how new information relates to what they already know. For example, they may need to decide if a new treatment would be substantially better for them, maybe with reduced risks or adverse effects, compared with their current options.

The aim of this project is 1) to explore how people with MS assess and integrate health information into their lives according to their needs, experiences, preferences, and values, and 2) how documentation to support people to make the connection between health information and their needs, experiences, preferences and values can be provided in an online resource of evidence-based health information for people with MS, and their families.

## Methods

**Figure 1:**
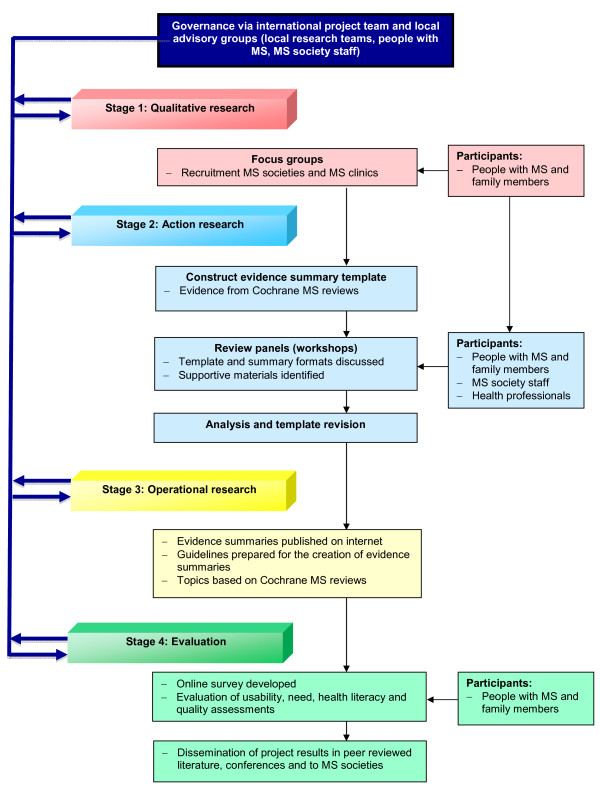
**Flow chart of IN-DEEP project**.

This project will employ a mixed methods approach involving four stages: qualitative research (Stage 1); action research (Stage 2); operational research (Stage 3); and evaluation (Stage 4) (see Figure 1). A mixed methods study combines and builds knowledge derived from qualitative and quantitative research methods [30]. It aims to generate theory and test it in latter stages.

The IN-DEEP project (Integrating and deriving evidence, experiences and preferences: Developing evidence-based health information applicable to decision making and self-management by people with MS) is a collaboration of project teams in Melbourne, Australia (led by SH) and Milan, Italy (led by GF). It will be conducted in parallel in Australia and Italy. The studies will be conducted as stand-alone projects but will follow the same stages and mixed methods approach. We anticipate some methodological differences between the projects as we progress due to differing findings from early research stages and contextual differences between countries. A comparison of similarities and differences in project findings between Australia and Italy will be considered at each stage and will be reported.

### Advisory groups

Prior to the commencement of the research, an Italian and an Australian advisory group will be formed. The advisory groups at each site will play key roles in guiding all project stages and will include members of the local research teams, local MS society staff (MS Australia in Australia and AISM in Italy), and people with MS. Academic-community partnerships benefit from the different perspectives and organisational styles [31]. Additionally, there is strong evidence that involving patients in the development of health information leads to information that is more understandable [32].

### Participants

#### People with MS, and family members of people with MS

People with MS, and family members (partners, parents, children and siblings aged over 18) of people with MS will be included in the study. It is often the case that close family members may be as or more actively involved in finding and assessing health information as the person with MS. To reflect the gender and age distribution of people with MS, we will aim to include women with MS in an approximate 3:1 ratio to men, and include participants in the age range of 20-69 [17]. Individuals outside that range (over 18 years) if they are interested to participate will also be accepted. A mix of people newly diagnosed with MS (up to three years since diagnosis), those with later stages of MS (living with MS for at least 4 years), and people taking immunotherapy medications will also be sought in order to capture a broad range of experiences. People with MS and family members of people with MS will be invited to take part in all four stages of the research.

#### MS society staff and health professionals

As well as including people with MS and their family members, Stages 2 and 3 will also include clinicians involved with MS treatment, and MS society staff who work in a health information capacity. The involvement of participants from a variety of perspectives means that the research can gain insight into a range of potential barriers to uptake of information from an online resource, and then be able to take steps to reduce these barriers in practice.

### Participant sampling

Purposive sampling will be used both in Australia and Italy. People with MS, family members, and health professionals will be recruited using the communication channels of MS Australia in Australia, and AISM in Italy and their networks of related organisations. In Australia, snowball sampling from the pool of people with MS and family members will also be undertaken, while in Italy collaborating neurologists will identify eligible patients with MS and family members. From these pools of participants, selected participants will be invited to take part in focus groups and the successive stages of the study.

### Research activities

#### Stage 1: Qualitative research

The aim of Stage 1 is to document and analyse the needs, experiences, preferences, and values of people with MS when assessing and integrating evidence-based health information into their decision-making about, and management of, their health. Between four and six focus groups will be conducted in each country. In Australia, focus groups will be held in Victoria and Tasmania, with at least one in a non-metropolitan area. In Italy, focus groups will be conducted in Northern, Central and Southern Italy. Where possible, groups will be stratified according to length of time since diagnosis and whether or not participants are a person with MS or a family member. The focus groups will last approximately one hour and will be conducted by two experienced researchers. The focus groups will be audio-recorded and transcribed verbatim.

Focus group topics will be based on four main questions: (1) Where do you get reliable information about the evidence of the effectiveness of treatments for MS?; (2) What kinds of information do you need, and how do these needs change over time?; (3) How do you use the Internet to access information about treatments for MS?; and (4) How do you assess the quality and usefulness of this information?

The focus group transcripts will be analysed by thematic analysis. Two researchers will independently read the transcripts multiple times and identify emerging themes. Agreed codes will be used to categorise the themes.

#### Stage 2: Action research

The aim of Stage 2 is to use the data from Stage 1 to generate a model that will guide the study team to develop evidence-based health information that is meaningful for people with MS and their family members, and that people can apply to their own situations. To do this, we will develop a template for summarising and presenting evidence-based health information (evidence summaries), which will be connected to supporting documentation to help people understand the summarised evidence, apply it to their personal circumstances, and use it when making health-related decisions. The exact nature of the evidence summaries and supporting documents will be fine-tuned in workshops (review panels) with people with MS, family members, health professionals and MS Society staff. Action research is used when the aim of the study is to both generate knowledge about a social system and, at the same time, attempt to change that system [33].

The evidence summaries will be created using a selection of systematic reviews of MS intervention effectiveness from *The Cochrane Library*. As at Issue 1, 2012 of The Cochrane Library, there were 36 published reviews about MS interventions, including reviews of medicines (such as corticosteroids and interferons) and other interventions (such as rehabilitation, diet, and allied therapies). These reviews are coordinated by the MS Cochrane Review Group, led by GF as the Coordinating Editor. Summaries will be prepared in English and Italian. Consideration will be given to information needs that are not met by Cochrane reviews.

The evidence summaries and supporting materials will be developed based on methods for presenting evidence to patients that have been identified in the literature. These summaries will be integrated with supporting documentation that is relevant to understanding evidence and to personal decision making, and will be informed by the themes that arose in Stage 1.

Two review panels per country will be held. The review panels will operate like focus groups but will have an element of 'review', which means a range of formats for presenting health information can emerge and be discussed and debated. The results of the panel meetings will be analysed by examining the themes discussed, participant comments, areas of agreement, and decisions made. The results will be discussed by the project team, and taken to the advisory group for consideration. The project team will consider any divergence between the Australian and Italian panels and the implications of these for the online resource.

#### Stage 3: Operational research

The aim of Stage 3 is to establish a model for presenting evidence-based health information (the evidence summaries and supporting documentation developed in Stage 2) on the Internet. This stage involves undertaking operational research, which is a method for linking research to practice, and for examining processes and outcomes for implementation [28,34].

The project team and advisory group will model, plan and create the online information resource, using the outputs of Stages 1 and 2. A protocol and guidelines for how the project team, MS society staff and people with MS can contribute to the development and maintenance of the documents for the online resource will also be created.

#### Stage 4: Evaluation

The aim of Stage 4 is to evaluate whether or not the IN-DEEP online resource meets the needs of people with MS and the family members of people with MS, according to the information gathered in Stage 1. In broad terms, the evaluation will undertake usability, need, health literacy, and quality assessments. This means the evaluation will assess people's experience of using the online health information resource; verify whether or not the information on the web site fulfils people's information needs; determine if people can understand and plan to use the information when making health-related decisions; and find out if people consider the information to be of good quality.

For the evaluation we will review the IN-DEEP project intended outcomes; develop an evaluation framework to summarise the project expectations and outline the steps of the evaluation; document deviations from the initial protocol; develop and pre-test an online questionnaire using evidence-based techniques; provide the final questionnaire for online use; analyse the results of the questionnaire; and report on the evaluation findings. An online questionnaire represents a low-cost but effective method for obtaining evaluation data [35]. Additionally, Internet-tracking tools will be utilised to keep a record of the number of 'hits' on the site. The evaluation results will be used to make recommendations to improve the online resource.

### Ethical considerations

Written, informed consent will be sought from all participants prior to their taking part in the study. Ethical approval has been granted by the Faculty of Health Sciences Human Research Ethics Committee of La Trobe University, Australia, and the Ethics Committee of the Fondazione IRCCS Istituto Neurologico "Carlo Besta", Italy.

## Discussion

The IN-DEEP project will develop a sound and theoretically informed online model that makes evidence-based health information accessible and understandable for people with MS and family members. The evidence-based research information will be supported by documentation that is reflective of people's needs, experiences, preferences and values, and will help people to integrate the research information into, and apply it to, their own circumstances.

The lack of carefully grounded consultations when developing health information has been a major gap in this area in the past [36]. The consultations for the IN-DEEP project will involve two main dimensions: the first is to undergo consultations with people with MS, their families, and health professionals associated with MS in order to identify the perceived gaps in information for treatments and self-management; and the second is to identify ways in which evidence-based research can be presented on the Internet to support people to make health-related decisions about treatments and self-management options.

This research will be cognisant of the challenges that people with low health literacy have. Health literacy encompasses ideas around a person's ability to access, understand and use health information relevant to their situation [8]. Australian research about the experience of living with MS illuminates a wider context for the need to make health information accessible and understandable. MS has pervasive and disabling effects on a person's life and that of their family [17,37]. Research into the support needs for someone with MS highlights the variable and ongoing nature of the demands placed on individuals and their families [38]. Individuals' stoicism and coping strategies need to be recognised and supported so that people can plan for and manage their health, and communicate their problems to family and health professionals. By making health information more understandable (evidence summaries) and accessible (available on the Internet), the IN-DEEP project seeks to increase the confidence of people with MS to access, understand and use new research and information about MS management and treatment that they may come across in the media or on the Internet. The IN-DEEP online health-information resource will provide accessible information about the effects of clinical, educational and behavioural interventions for MS.

An important aspect of the IN-DEEP research is the opportunity to compare differences between two countries when developing health-related information and the platform from which to provide that information to consumers. Routinely, research summaries are translated verbatim where possible for publication on non-English web sites. The IN-DEEP research will enable us to examine if there are key differences between English and non-English versions of health-related information that could inform international practice.

This research is at the forefront of international efforts to improve the quality and relevance of evidence-based information for use by people with MS, and to patients and their families/carers more widely. People with MS are not only users of health information, they are future drivers of policies for a more responsive health care system [39]. Due to the demands of illness, people with MS and their families have to become skilled users of information, and able to find support when needed. In recognition of this, this project will involve people with MS, their families, health professionals and non-government organisations in a partnership model for the co-production of new knowledge and resources [40].

## Abbreviations

MS: (multiple sclerosis); AISM: (Associazione Italiana Sclerosi Multipla).

## Competing interests

The authors declare that they have no competing interests.

## Authors' contributions

SH led the conceptualisation and design of the project, prepared the original (Australian) project proposal and obtained funding. GF prepared the original (Italian) project proposal and obtained funding. MS, DB, PM, CC, SH, RO contributed to all these processes. SH will oversee the Australian project as principal investigator. AS will coordinate data collection, template development, analysis, interpretation, report writing and dissemination with input from MS, SS, RO, MH and the Italian team. GF will oversee the Italian project as principal investigator. CC and DB will coordinate data collection, template development, analysis, interpretation and dissemination with input from PM, MAB and the Australian team. AS and SH developed this manuscript from the Australian project proposal, GF and CC from the Italian project. All authors read and approved the final version of the manuscript.

## Pre-publication history

The pre-publication history for this paper can be accessed here:

http://www.biomedcentral.com/1472-6947/12/20/prepub
